# Family Drawings before and after Treatment for Child Conduct Problems: Fluidity of Family Dysfunction

**DOI:** 10.1007/s10826-017-0841-2

**Published:** 2017-07-31

**Authors:** Lilian Kloft, David Hawes, Caroline Moul, Sonia Sultan, Mark Dadds

**Affiliations:** 1 Faculty of Psychology and Neuroscience, Maastricht University, Maastricht, The Netherlands; 2 School of Psychology, University of Sydney, Sydney, Australia

**Keywords:** Parent training, Conduct problems, Callous-unemotional traits, Family functioning, Family drawings

## Abstract

Children’s drawings have previously been found to reflect their representations of family relationships. The present study examined whether evidence-based parent training for child conduct problems impacts on representations of family functioning using the Family Drawing Paradigm (FDP). *N* = 53 clinic-referred children (aged 3–15) with conduct problems and their families were assessed pre-treatment and at 6-month follow-up on a modified version of the FDP. Analyses of changes in the FDP revealed improvements in family functioning but not tone of language (as indicated by written descriptors) following treatment. Higher family dysfunction scores were associated with increased levels of callous-unemotional (CU) traits in the children pre-treatment. Children with high levels of CU, however, demonstrated greater change in FDP dysfunction than a low CU group, resulting in similar levels at follow-up. CU traits also moderated the association between change in family warmth and conduct problem severity, with increased FDP warmth more strongly related to improved conduct problems in the high vs. the low CU group. FDP drawings are sensitive to changes in family functioning arising from parent training, accounting for unique variance in child outcomes independent of verbal reports.

Children with early-onset conduct problems (CPs) are at high risk for chronic antisocial and aggressive behaviour, and a variety of social and mental health problems in adolescence and adulthood (e.g. delinquency, psychiatric disorders, substance use, school dropout; Fergusson et al. 2005; Kratzer and Hodgins 1997). Research conducted over the past four decades has, however, shown that conduct problems can be treated successfully (Beauchaine et al. 2005). Among the various treatment models currently available, parent training interventions based on social learning theory are regarded as particularly well-established and efficacious. In these programs parents are coached in behavioural strategies for increasing reinforcement of adaptive child behaviour and setting consistent limits on disruptive behaviour, thereby replacing escalating cycles of parent-child coercion with positive, relationship-enhancing interactions (Hawes and Allen 2016). The intervention’s large evidence base demonstrates clinically significant improvements for typically about two thirds of participant children, including short- and long-term benefits such as reduced disruptive behaviour and improved parental mental health (e.g. Beauchaine et al. 2005; Webster-Stratton et al. 1989).

Despite their well-established effectiveness, there remains a need to examine how parenting interventions produce change, that is, identify the mechanism(s) through which treatments operate (Kazdin and Nock 2003). Traditionally, parent training programs target parenting practices in order to improve the effectiveness of discipline and the quality of parent-child relationships. Accordingly, there is considerable evidence that improvements in parenting skills are key mechanisms of change in these interventions, with outcomes accounted for by both increased positive parenting (e.g., warmth, contingent positive reinforcement) and decreased negative parenting (e.g., harsh/inconsistent discipline; Beauchaine et al. 2005; Gardner et al. 2006). Some intervention programs also include components targeting broader family functioning (e.g., marital relationship, behaviour of siblings and other family members), and research has supported that these broader family dynamics change in response to treatment. For example, Adams (2001) found that parents who participated in a parent training program reported healthier family functioning in the areas of problem solving, communication, affective responsiveness, and behaviour control, compared to families who received regular mental health services. In observational research examining changes in parent-child interaction from pre- to post-treatment it has also been found that the families of children who benefit from such treatment show increased emotional flexibility in problem-solving discussions (Granic et al. 2007).

Although an improvement in parenting practices appears to be the key mechanism of change, research to date into treatment mechanisms has been limited by a reliance on parent-report and observational methods, and has neglected data on the child- and family process-levels. There are, however, various methods available that can reliably measure children’s representations of family relationships. A representational measurement technique that is readily usable in clinical settings and can easily be adapted as a family collaborative task is the use of family drawings. Drawing is a common and natural activity for children, and its nonverbal nature has the advantage of offering the possibility to express conscious as well as unconscious representations of family characteristics (Fury et al. 1997).

There is growing support for the use of children’s drawings as a valuable method to assess children’s needs, attitudes and conflicts. Studies have supported the use of children’s drawings of their family to understand individual differences in child and family functioning (e.g. Leon et al. 2007; Roe et al. 2006), wherein the embellishment, detail, vibrancy, size and position of figures can reflect children’s internalizations of caregiving experiences (Burkitt et al. 2003). These studies commonly use the Family Drawing Paradigm (FDP; Fury et al. 1997), a standardized tool developed to assess internal representations of family functioning that has been extensively applied and validated within a range of diverse racial ethnic and international samples (e.g., Behrens and Kaplan 2011; Goldner and Scharf 2011). A study by Purvis and Cross (2006) compared at-risk adopted children’s family drawings before and after a therapeutic day-camp and found improvements in attachment representations. However, no study to date has looked at family drawings done not by children alone but in collaboration with their families in an intervention context.

Knowledge of mechanisms of change in parent training programs may be particularly important given poor treatment outcomes for a proportion of children, suggesting that distinct mechanisms of change might operate in distinct subgroups of families referred for child conduct problems. Researchers have endeavoured to identify variables affecting treatment response, and a host of factors have been found to predict poor outcomes, most of which are parental or family variables (e.g. parental psychopathology, marital conflict, socioeconomic disadvantage; Beauchaine et al. 2005). More recently, studies have focused on child factors and a consistent finding has been that behavioural parent training appears to be less effective in treating conduct problems in children with high callous-unemotional (CU) traits (e.g., limited empathy and guilt, shallow affect; Hawes et al. 2014). Levels of CU traits moderate the link between conduct problems and parenting practices such that ineffective parenting practices such as harsh or inconsistent discipline are more strongly related to conduct problems of boys with low rather than high CU traits. For children with high CU traits, the affective quality of the parent-child relationship (as opposed to parental discipline) is more strongly related to their conduct problems (Kochanska 1997; Pasalich et al. 2011). Children with high CU traits may be particularly sensitive to the influence of warmth in the parent-child relationship for the development of emotional responding (e.g., empathic concern) and internalization of parental moral and rule-based values, both of which protect against future antisocial behaviour (Hastings et al. 2000; Kochanska et al. 2008).

In the one existing study to address these issues using family drawings, Wagner et al. (2015) investigated associations among early parenting, conduct problems and callous-unemotional behaviours, and children’s representations of family relationships measured by the FDP in a large prospective study. It was found that greater dysfunctional representations were significantly associated with higher CU behaviours but not conduct problems, and that dysfunctional family representations partially accounted for the link between sensitive parenting and later CU behaviours, indicating that the internalization of caregiving may be one of multiple developmental mechanisms contributing to the association between parenting and callous-unemotional conduct. This study indicates that use of the FDP may offer an innovative way to assess change, and the mechanisms of change, in family dynamics associated with parenting programs for child conduct problems.

Building on the research by Wagner et al. (2015), the current study assessed whether changes in family drawings reveal important mechanisms of change associated with family dynamics in parents and children undergoing treatment for child conduct problems. Previous applications of the FDP were child-focused with family drawings done by the target child separated from the family; however, in the present study the drawing task was modified in two ways: (1) families were instructed that everyone was allowed to contribute to the drawing, and (2) the family needed to discuss and agree on which colour and one-word descriptor would be used to describe each family member. Thus, the FDP was used as a family task designed to provoke discussion and to reach family ‘consensus’, following in the footsteps of other family interaction studies which have a long history in psychiatry (e.g., Doane 1978; Wahlberg et al. 2004). Using a prospective within-subjects design, the current study investigated changes in families’ representations of family functioning in response to an evidence-based parent training intervention, assessed through the modified Family Drawing Paradigm at pre-treatment and 6-month follow-up. Based on extant literature it was expected that: (a) families’ drawings would show less family dysfunction as measured by the FDP following treatment compared to before the intervention. Based on the observations of Wagner et al. (2015) it was hypothesized that: (b) greater representations of family dysfunction would be significantly associated with child CU traits. As affective relationship quality is especially important to behavioural change in children with high CU traits, an association between change in warmth in the pictures and child outcome would be moderated by levels of CU traits, such that more warmth would be more strongly related to positive outcomes in the child for children with high CU traits compared to those with low CU traits (hypothesis c). On the other hand, change in family organization or cooperation would be more strongly related to change in child outcomes for children with low CU traits (hypothesis d). Finally, it was also hypothesized that: (e) the written one-word descriptors of family members would be more positive after compared to before treatment.

## Method

### Participants

Participants were families with children self-referred or referred from schools or mental health professionals for child externalizing behaviour and emotional problems to the University of New South Wales (UNSW) Child Behaviour Research Clinic (CBRC) in Sydney, Australia, between 2007 and 2015. Inclusion criteria included referral to the clinic for conduct problems, child age (from 3 to 15 years), IQ (>70), no major illness, participation in three or more treatment sessions, and completion of the family drawing both pre- and post-intervention. A total of 52 families with children aged 3–15 (*M* = 6.3, SD = 2.6) years were included in the study. One family with twins who were both referred was included, resulting in a total of 53 target children. The sample included 14 (26%) female and 39 (74%) male children, with this gender distribution comparable to that found in previous samples in this area of study (Hawes et al. 2013; Kolko and Pardini 2010). While all children were referred for treatment of conduct problems, comorbidity in such samples is common and conduct problems tend to occur in context of various diagnoses. A total of 27 children (51%) were diagnosed with a primary disruptive behavioural disorder, i.e., oppositional defiant disorder (ODD) or conduct disorder (CD), while five (9%) received a primary diagnosis of attention deficit hyperactivity disorder (ADHD), five (9%) received a primary anxiety/mood disorder diagnosis, and two children (4%) were given a primary autistic spectrum disorder (ASD) diagnosis. Occurrences of diagnoses anywhere in the profile were: ODD/CD 62%, ADHD 25%, anxiety/mood disorder 13%, ASD 6%. In total, 45% of children received one diagnosis, 30% were given two diagnoses, while 25% did not receive a formal diagnosis, but presented with sub-clinical symptoms. All diagnoses were based on the DSM-IV criteria (American Psychiatric Association 1994) by the treating clinician during an initial assessment with parents, using the Diagnostic Interview Schedule for Children, Adolescents, and Parents (DISCAP; Holland and Dadds 1997). Interrater reliability among a team of psychologists/psychiatrists for DISCAP diagnoses was good (average Cohen’s *κ* = .74) Participants in receipt of stimulant medication for behavioural problems (at either pre-treatment or follow-up) represented 6% of the overall sample.

Caregivers were both biological parents in 38 families (73%), one biological parent in 8 families (15%), one biological parent and a partner in two families (4%), a guardian in one family (2%), shared care among biological parents and partner/s in one family (2%), and grandparent/s in two families (4%). Marital statuses were as follows: married 73%, de facto 8%, separated 9%, divorced 2%, single 4%, other 4%. On average, families had 2.3 children (SD = 1.03). Parent’s highest education level ranged from: 4 years of secondary school (mothers: 6%, fathers: 12%), to 6 years of secondary school (mothers: 6%, fathers: 10%), to technical/skills-based tertiary education (mothers: 23%, fathers: 19%), to university education (mothers: 63%, fathers: 54%; 4 missing values in total).

### Procedures

Permission to conduct this research was obtained from the University of New South Wales Human Research Ethics Committee, and informed consent provided by parents at the time of recruitment. Parents and children completed all measures at time 1 as part of pre-clinical assessment. Participants were reassessed for diagnostic severity and representations of family dysfunction 6 months following the completion of treatment (time 2).

All families received treatment consisting of a fully manualised, 10-week, social-learning based parent-training programme by Dadds and Hawes (2006). This evidence-based program trains parents in strategies for effectively reinforcing positive child behavior (e.g., descriptive praise), and setting limits on antisocial behavior (e.g., time-out). These child management strategies are supplemented with components that target parent/systemic issues impacting on child adjustment (e.g., relationship discord, parental stress), and individual child-focused sessions may be added. Thus, length of treatment is in part determined by the needs of the individual family (e.g., inclusion of additional modules). The intervention commenced with a 1.5-h assessment session which all core family members were asked to attend, followed by a child observation session and 9 weekly 1-hr sessions. The content of the sessions is as follows: session 1 consists of a parent interview, session 2 features additional assessment (e.g., parent-child observation; child interview), session 3 introduces core change strategies (responding to good behaviour, responding to misbehaviour), session 4 functions as a review of strategies, session 5 introduces advanced strategies (e.g., managing sibling conflict, high-risk situations), session 6 is another review, sessions 7 and 8 focus on parent care, and session 9 is a module for review and relapse prevention. Interventions were delivered on an individual basis with families, by psychologists with at least 1 year experience in the treatment of children and families. The average number of sessions attended by parents was 5.45 (SD = 1.67). Cases were discussed in weekly supervision meetings attended by therapists on the project to ensure that treatment integrity was maintained.

### Measures

#### Families’ representations of family dysfunction

As part of the first assessment session, families (i.e., all family members present in the session) were asked to complete a drawing of their family. They were instructed that anyone can participate in doing the drawing, and that one word should be assigned to each family member to describe that person. Family members were instructed to agree on the colours used and on the descriptors chosen for each family member. They received either an A4 or A3 sheet of blank paper and a set of 8–10 either coloured pencils or felt-tip pens. The task was afforded ten minutes and drawings were labelled with regard to the family ID number and the session (pre vs. 6-month) by the clinician conducting the task. Prior to coding the drawings in accordance with the Family Drawing Paradigm (FDP; Fury et al. 1997), all drawings were scanned by a research assistant not involved in the coding process who digitally black-marked all one-word descriptors and all indications of session on the scanned drawings. This was done in order to separate the measures of family dysfunction (drawing) and tone of language (descriptors), resulting in pure and unbiased measures. Additionally, this enabled the coder to remain blind with regard to the drawing being a pre- or post-treatment drawing, thus avoiding bias.

The FDP was coded using the global rating scales originally developed by Kaplan and Main (1986) and adapted by Fury et al. (1997). An adaptation of these rating scales was previously used in a series of studies conducted as part of the Family Life Project (e.g., Zvara et al. 2014), and the current coding protocols are based on this system but were further adapted to suit our version of the drawing task. Specifically, in addition to the original scales (Vitality, Family Pride, Vulnerability, Emotional Distance, Tension/Anger, Role Reversal, Bizarreness, Global Pathology), the scales Parental Team and Role Exaggeration were developed. Moreover, the Family Pride, Emotional Distance, and Tension/Anger scales were adjusted to capture family dynamics not limited to the target child. Hence, the current analyses use ten 5-point rating scales (ranging from “very low” to “very high”) to compute a latent factor labelled representation of family dysfunction, where higher scores signal higher levels of dysfunction.

**Fig. 1:**
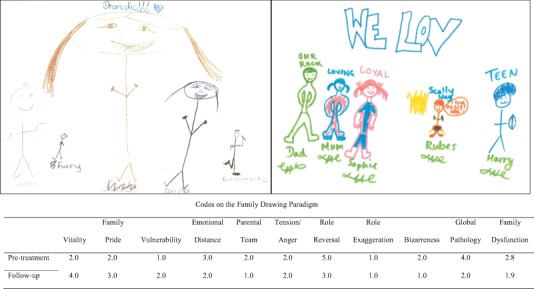
Example of a family drawing at pre-treatment (*left*) and 6-month follow-up (*right*) of one participating family. Respective scores on the Family Drawing Paradigm are depicted below

Vitality indicates the emotional investment in the drawing as reflected in embellishment, detail, vibrancy and creativity. Family Pride is evidenced by positive facial affect across figures, and commonality, context, or proximity among family members. These two first scales are reverse scored. Vulnerability is inferred from size distortions, placement of figures, and negative affect or closed body positions. Emotional Distance is based on spatial distance between caregivers and children, neutral or negative affect, closed body positions, and barriers indicating separation. Parental Team focuses on emotional distance between caregivers (only coded for families in which both parents are caregivers) and has the same criteria as the Emotional Distance scale. Tension/Anger is observed from rough drawing, scribbling, and carelessness in terms of effort and fine details. Role Reversal is signified by size distortions with the target child or siblings drawn larger than the caregivers. Role Exaggeration is inferred from size distortions where the caregivers are proportionally enlarged. Bizarreness is expressed by unusual signs, symbols or fantasy themes in the drawing. Global Pathology reflects the overall degree of negativity of the drawing, considering all other scale-scores as well as elements not easily classifiable with the prior scales. The FDP has previously been found to have good predictive validity (e.g. Zvara et al. 2014). An example of one family’s pre- and post-treatment drawings can be viewed in Fig. 1.

Given the modification of the FDP into a task involving contribution by multiple people, coders were asked to estimate the content drawn by a child vs. an adult. Knowing whether a drawing was mostly done by a child or an adult provides important information about family dynamics and is essential to the interpretation of effects.

#### Family disorganization

A 5-point scale (ranging from “very low” to “very high”) was constructed to assess family disorganization. Family disorganization is signalled by the extent to which the family correctly followed the instructions given for the drawing task (i.e., assigned a one-word descriptor to each family member), any errors made (crossed-out words or drawings), and the extent to which the drawing was complete (all family members fully drawn and none missing).

#### One-word descriptors

The valence of the one-word descriptors was assessed by first dividing all descriptors manually into positive, negative, and neutral word categories. An index was then computed indicating the overall degree of positive word content of a drawing that was standardized for the number of family members (ranging from −1 to 1 with higher numbers indexing more positive tones). This index will subsequently be referred to as the drawing’s ‘tone of language’. Further, descriptors were divided among the categories of Target Child, Mother, Father, Siblings, and Other (i.e., pets, other family members) according to their labelling in the drawings.

#### FDP warmth

The Emotional Distance scale of the Family Drawing Paradigm was taken as an indicator of family warmth.

#### Conduct problems

Severity of behavioural problems on the Diagnostic Interview Schedule for Children, Adolescents, and Parents (DISCAP; Holland and Dadds 1997) was used as a continuous measure of conduct problems. Interrater agreement was calculated using bivariate correlations between diagnosticians (*r* = .70). Conduct problems at time 2 was regarded as an index of child outcome.

#### Callous-unemotional traits

CU traits were measured using the UNSW system developed by Dadds et al. (2005) of combining items from both the Antisocial Process Screening Device (Frick and Hare 2001) and the Strengths and Difficulties Questionnaire (Goodman 1997). This scale has been comprehensively validated in research into CU traits (e.g., Dadds et al. 2005, 2009). Multi-informant data from parents and teachers on CU traits was collected; however, analyses relied on mother reports due to higher availability of CU trait ratings (mothers 100%, fathers 75%, teachers 79%). Mother and father CU reports were positively correlated as were mother and teacher reports (*r* = .32 and *r* = .30 respectively, all *p*s < .06), indicating moderate convergence.

Participants were divided into two groups of high and low CU traits using the standard cut-off by Dadds et al. (2014), classifying children as high in CU traits if their score was 8 or greater. This resulted in group sizes of *n* = 33 (62%) for the low scoring and *n* = 20 (38%) for the high scoring children. Dichotomization of CU traits scores into high and low groups is well-established and empirically supported practice in the field (Vasey et al. 2005), and the cut-off of 8 or greater is well validated using the current measure of CU traits (e.g. Dadds et al. 2014).

#### Additional measures

In order to assess Child IQ, the Wechsler Intelligence Scale for Children (WISC-IV), the Wechsler Preschool and Primary Scales of Intelligence (WPPSI-III), or the WebNeuro (Silverstein et al. 2007), were administered, depending on the child’s age. A standardized index was then created combining scores of the aforementioned tests to form an estimate of intellectual functioning. The Depression Anxiety Stress Scales-42 (DASS-42) is a self-report measure of negative emotional states (Lovibond and Lovibond 1995), and was completed by parents. The DASS-42 has proven its reliability and validity in the assessment of depression, anxiety, and stress (Antony et al. 1998).

### Data Analyses

First, reliability analyses were conducted for the study’s main variables. Next, analyses on child vs. adult involvement in the family drawings were conducted. The possibility of confounding variables was explored by testing correlations between representations of family dysfunction, family demographics, and other child and parent adjustment variables. Similarly, equivalence of the high and low CU groups was tested by performing analyses of variance on key variables (e.g., child adjustment, number of treatment sessions). Variables found to be significantly associated with family dysfunction or CU groups were entered as covariates in further analyses.

Finally, the proposed hypotheses were tested. A paired-samples t-test was used to test for differences in total family dysfunction scores over time (hypothesis a). Differences between the high and low CU traits groups in overall family dysfunction (hypothesis b) were tested using one-way analyses of variance. Further, multivariate analyses of variance (MANOVA) were employed to test effects of time (as a repeated factor) and CU traits (as a moderator) on all other dependent measures (FDP scales, family disorganization, tone of language). This allowed following up on hypotheses a and b. Next, moderation effects of CU traits on associations between child outcome and change in emotional distance (hypothesis c) and change in family disorganization (hypothesis d) were tested using Generalized Estimating Equations (GEE). This procedure extends the general linear model to allow for analysis of repeated measurements. We also used GEE to clarify the relationship between CU traits, change in family dysfunction, and change in conduct problems. Lastly, differences in family tone of language (hypothesis e) were analysed using paired-samples t-tests, given that MANOVA procedures were inept due to high numbers of missing data.

## Results

### Reliability analyses

#### Family drawing paradigm

Forty six percent of all drawings were double-coded by two independent coders trained to reliability, and the primary coder’s scores were used for the final analyses. Interrater reliabilities were calculated using bivariate correlations which ranged from *r* = .58 to *r* = .78 (*M* = .70; SD = .07), indicating high overall interrater reliability. The internal consistency for the family dysfunction score was good with Cronbach’s *α* = .67 for time 1 and Cronbach’s *α* = .74 for time 2. In addition, all coders were asked to estimate the percentage of content drawn by a child. Interrater reliabilities were calculated using correlations and were *r* = .63 at time 1 and *r* = .57 at time 2 (*p* < .001).

#### Family disorganization

Interrater reliability was high with interrater data correlating *r* = .74.

#### Tone of language

For reliability purposes, all one-word descriptors were double-coded (*n* = 166). There was 88.5% agreement and the interrater reliability for the two raters was found to be kappa = .82. In case of discrepancy, the codes were conferenced after calculating the reported interrater reliability. Moreover, the final categorisations were compared to Hu and Liu’s sentiment lexicon lists of positive and negative words containing approximately 6800 words, which have been compiled over several years and are used for sentiment and opinion analysis (Hu and Liu 2004; Liu et al. 2005). There was 81.6% agreement among the categorisations, and an interrater reliability analysis resulted in a kappa = .72, indicating substantial agreement.

In order to investigate whether the descriptors coincided with representations of family dysfunction in the pictures, relationships between tone of language and the FDP variables were inspected. Significant correlations were found for tone of language at time 2 with the Vitality (*r* = −.28), Role Reversal (*r* = −.32), and Global Pathology scales (*r* = −.36), but not at time 1. This indicates moderate convergence of tone of language and important aspects of family dysfunction at time 2 (inverse relationship), but no convergence at time 1.

### Child vs. adult involvement

As the FDP was modified to allow family involvement, we looked at coders’ estimates of the percentages of content drawn by a child vs. an adult, comparing estimates at time 1 and time 2. The estimated content of the drawings attributable to a child was found 79% on average or greater. There was a borderline significant trend for a higher percentage of content drawn by a child at time 2 (*M* = 87.40%) relative to time 1 (*M* = 79.23%; *p* = .051). No associations of percentage of content drawn by a child were found with family dysfunction at time 1 or time 2, change in family dysfunction, CU traits, or child outcome. Thus, varying levels of child or adult responsibility for the drawings did not confound other effects tested in this study and did not need to be included as a covariate in further analyses.

### FDP and child and family variables

Associations of overall family dysfunction at time 1 with age, gender, child IQ, level of household income, number of siblings, mother’s and father’s level of education, and mother’s and father’s age (time 1) were tested. No significant associations were detected. Data on child IQ were missing in 25% of cases. Associations of FDP family dysfunction at time 1 with symptom severity on the diagnostic dimensions of the DISCAP were tested next. No significant associations were found between the overall family dysfunction score and ODD/CD, ADHD, and anxiety/mood disorder symptom severity. However, ASD symptoms were found to be negatively associated with family dysfunction at time 1 (*r* = −.30). As only two children within the sample presented with high ASD symptoms, these two cases were excluded from further analysis. These data are depicted in Table 1.

**Table 1:** Correlation matrix for overall FDP family dysfunction and child/family variables (time 1)

Variables	Age	Gender	IQ	Number of siblings	Household income	Education level mother	Education level father
Family dysfunction	.03	.01	−.02	−.04	.3	.14	.13
Variables	Age mother	Age father	ODD/ CD	ADHD	ASD	Anxiety/mood disorder	
Family dysfunction	−.01	−.05	.17	.09	.30*	.02	

*p < .05; **p < .01

As child adjustment is intimately related to parental psychopathology, we also examined relationships of the FDP subscales to mother’s and father’s reports on the Depression Anxiety Stress Scales (DASS), both measured at time 1. Correlations can be inspected in Table 2. When interpreting the correlations, it should be borne in mind that a higher score on an FDP scale signifies a higher degree of dysfunction on that scale. With regard to mother’s reports, there were significant positive associations of the Family Pride scale and depression (*r* = .34), and Role Reversal and anxiety (*r* = .37). For father’s reports, depression positively correlated with Role Exaggeration (*r* = .40), and anxiety negatively correlated with Tension/Anger (*r* = −.36). On the whole, however, only four of 60 possible correlations were significant, and so dysfunction in the family drawings was largely unrelated to parental anxiety, depression and stress.

**Table 2:** Correlation matrix for the family drawing paradigm scales and the depression anxiety stress scales

	Mother Reports			Father reports		
Variables	Depression	Anxiety	Stress	Depression	Anxiety	Stress
Vitality	−.16	.17	−.2	−.22	−.3	−.29
Family pride	.34*	.19	.18	.06	−.3	−.08
Vulnerability	.22	−.09	.13	.03	−.14	−.04
Emotional distance	.22	.14	.08	−.01	−.15	−.14
Parental team	.23	−.05	.12	.06	.05	−.12
Tension/anger	−.03	−.22	−.2	−.03	−.36*	−.28
Role reversal	.18	.37**	.23	−.03	−.02	.12
Role exaggeration	−.05	−.19	.11	.40*	.09	.19
Bizarreness	−.07	−.19	−.14	.21	−.12	−.05
Global pathology	.14	.12	.1	.01	−.2	−.23

*p < .05; **p < .01

### Equivalence of CU groups

ANOVA tests revealed no significant group differences for CU traits on the variables of age, symptom severity on any of the DSM-IV diagnostic dimensions at time 1, and number of treatment sessions attended by parents.

### Hypotheses Tests

For the overall sample, there was a significant difference in overall family dysfunction scores over time (*t* = 3.24, *p* *<* .01), with lower dysfunction scores at time 2 (*M* = 2.14; SD = .53), compared to time 1 (*M* = 2.41; SD = .51). This indicates that representations of family dysfunction decreased over time, and supports our main hypothesis (a).

**Fig. 2:**
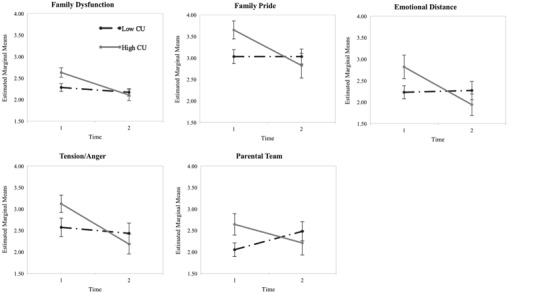
Interaction plots for callous-unemotional traits and family dysfunction, family pride, emotional distance, tension/anger, and parental team over time with standard errors

Next, we tested differences in family dysfunction scores between high and low CU groups before and after treatment. The repeated measures ANOVA showed a main effect for Time, *F* (1,49) = 15.42, *p* < .01, no main effect for CU traits, but an interaction between Time and CU traits, *F* (1, 49) = 5.62, *p < *.05. Follow-up univariate analyses revealed a significant difference at time 1, *F* (1,49)* = *5.60, *p < *.05, but not time 2, *F* (1, 49)* = *.19, *p > *.10. Inspecting the mean scores it became apparent that before the intervention, the high CU group showed higher levels of representations of family dysfunction than the low CU group (*M* = 2.65, SD = .50, and *M* = 2.32, SD = .48, respectively), while at 6-month follow-up, their scores were rather similar (*M* = 2.12, SD = .57 and *M* = 2.19, SD = .51, respectively). This means that families with children who were classified as having high levels of CU traits started off with greater representations of family dysfunction, compared to families whose children scored low on CU traits, which partially supports our hypothesis b. A plot that depicts this development visually can be examined in Fig. 2.

Next, a multivariate analysis of variance was conducted in order to investigate differences in FDP family dysfunction in more depth, with all FDP subscales (except for Parental Team due to missing values) as well as Family Disorganization as dependent variables, Time as a within-subjects factor, and CU traits as a between-subjects factor. There was a significant main effect for Time on the combined dependent variables, Wilks’ Lambda = 0.61, *F* (10, 40) = 2.59, *p* *<* .05, partial eta squared = .36, reflecting a general improvement in family dysfunction scores. There was no significant main effect for CU traits, but a significant interaction was found for Time and CU traits, Wilks’ Lambda = 0.54, *F* = 3.35, *p < *.01, partial eta squared = .42.

When the effects of Time on the dependent variables were considered separately, the scales for Family Pride, Vulnerability, Emotional Distance, Tension/Anger, and Global Pathology showed a significant difference at *p < *.05, and the Role Exaggeration scale showed borderline significance (*p* = .06). An inspection of the mean scores revealed that scores on these Family Drawing Paradigm variables were lower (i.e., evidencing less dysfunction) at time 2 compared to time 1. These findings add further support to hypothesis a. The mean scores and SDs are displayed in Table 3.

**Table 3:** Mean scores and standard deviations for high and low callous-unemotional traits groups on the Family Drawing Paradigm scales at Time 1 and Time 2

	Time 1				Time 2			
	Low callous-unemotional traits		High callous-unemotional traits		Low callous-unemotional traits		High callous-unemotional traits	
Variables	*M*	SD	*M*	SD	*M*	SD	*M*	SD
Vitality	2.77	1.04	3.18	0.88	2.80	0.71	2.59	0.80
Family pride	3.03	0.89	3.65	0.86	3.03	0.96	2.82	1.19
Vulnerability	2.03	0.93	2.35	0.86	1.60	0.81	1.59	0.87
Emotional distance	2.23	0.82	2.82	1.13	2.27	1.17	1.94	1.03
Parental team	2.05	0.74	2.64	0.93	2.48	1.03	2.21	1.05
Tension/anger	2.57	1.17	3.12	0.99	2.43	1.10	2.18	0.95
Role reversal	2.07	1.20	2.53	1.23	2.20	1.19	2.35	1.17
Role exaggeration	1.70	1.28	1.35	0.86	1.50	1.08	1.00	0.00
Bizarreness	2.07	0.87	1.88	0.86	1.57	0.63	1.76	0.97
Global pathology	2.43	0.90	2.88	0.99	2.00	0.83	1.76	0.83
Family disorganization	2.53	1.11	2.35	1.27	2.30	1.02	2.12	0.86

When the Time × CU traits interaction effects were considered separately, significant differences were observed for the Family Pride, Emotional Distance, and Tension/Anger, scales (*p < *.05), suggesting that the high and low CU groups showed different responses across time on these scales. An inspection of the mean plots (see Fig. 2) indicated that the differences in scores over time for the low CU group was smaller than the differences in scores over time for the high CU group, suggesting that the high CU group demonstrated more change (i.e., improvement) on these scales with treatment.

Parental Team was tested as a dependent variable for families with two-parent families in a second analysis (*n* = 37). Inspecting the pairwise comparisons, no significant main effect of time was found for this variable, but a significant interaction effect emerged, Wilk’s Lambda = .41, *F* (11, 25) = 3.27, *p < *.05. A means plot showed that the high CU group’s dysfunction scores on this scale improved over time, whereas the scores of the low CU group deteriorated (see Fig. 2).

As reported above, significant Time and Time × CU traits interaction effects were found for Emotional Distance, but not Family Disorganization. This already provides some information about our hypotheses c and d; nevertheless, we tested both hypotheses utilizing Generalised Estimating Equation (GEE) analyses with conduct problems at time 2 as the dependent variable, CU traits as a between-groups factor, and conduct problems at time 1, change in Emotional Distance, and change in Family Disorganization, and the interactions between CU traits and change in emotional distance and family disorganisation, as covariates. The analyses were conducted with the sample split into high and low CU groups. Only conduct problems at time 1 and the interaction between change in Emotional Distance and CU traits were found to significantly predict the outcome variable of time 2 conduct problems (*p < *.05). In order to dismantle the interaction, a linear regression was conducted within each of the CU groups. Inspecting the standardized regression coefficients revealed that the relationships were of opposite direction: for the high CU group, change in Emotional Distance was negatively related to the outcome (*β* = −.31), while it was a positive association for the low CU group (*β* = .24). Thus, more improvement in Emotional Distance was associated with less conduct problems after the intervention for the high CU group, and vice versa for the low CU group. While no evidence in support of our hypothesis d was found, hypothesis c was therefore supported.

Given our findings indicating that the high CU group evidenced more improvement in dysfunction scores over time than the low CU group, we wanted to formally test moderation effects for CU traits and child outcomes. A GEE analysis was conducted with conduct problems at time 2 as the dependent variable, CU category as a factor, and change in family dysfunction, conduct problems at time 1, age, and the interaction of CU traits and family dysfunction change, as covariates. Age, family dysfunction change, and the interaction between CU category and family dysfunction change were found to significantly predict conduct problems at time 2 (*p < *.05). This indicates that CU traits group moderated the relationship between change in family dysfunction and conduct problems at time 2, and again, a linear regression was conducted to elucidate the directions of relationships. The standardized regression coefficients revealed that an improvement in family dysfunction scores was significantly related to outcome for the high CU traits group (*β* = −.42) but not the low CU group (*β* = .04).

#### Analysis of family tone of language

No significant difference over time was found for the overall tone of language index. When considering differences over time specific to the family member in question, no significant differences between time 1 and time 2 were detected either. Thus, family tone of language was not found to be more positive following the intervention, refuting hypothesis e. The means and standard deviations of these data can be inspected in Table 4.

**Table 4:** Mean scores and standard deviations of family tone of language variables

	Time 1			Time 2		
	*M*	SD	*n*	*M*	SD	*n*
Overall index	0.58	0.48	47	0.63	0.40	47
Target child	0.58	0.63	39	0.64	0.58	47
Mother	0.71	0.64	42	0.80	0.46	44
Father	0.70	0.62	37	0.50	0.75	34
Siblings	0.55	0.51	32	0.56	0.62	35
Other	0.20	0.77	9	0.30	0.82	15

*Notes*: Descriptors were coded as either positive (+1), neutral (0), or negative (−1)

## Discussion

This study examined the effects of an evidence-based parent-focused family intervention for child conduct problems on representations of family dysfunction as evidenced in family drawings in a sample of clinic-referred children with disruptive behaviour problems and their families. Using a unique shared drawing task, drawings were analysed in accordance with the Family Drawing Paradigm (Fury et al. 1997), and compared before and 6 months after the intervention. We looked at different aspects of family dysfunction, as well as the tone of family language, and differences between groups of children with high and low callous-unemotional traits. This study investigated children’s drawings in the context of a parent training intervention for conduct problems, and featured a new extension and application of the FDP to suit a family systems approach.

In line with our main hypothesis, we found overall that family dysfunction scores were significantly reduced following the parenting intervention. While this intervention is well established for producing positive change in parenting skills and confidence, parent–child interactions, broader family processes, and child behaviour problems, this study showed that such positive changes can be detected in drawings using the structured Family Drawing Paradigm. Although the lack of a control group limits the certainty with which we can attribute the changes to the intervention, no associations with age or IQ were detected, thereby indicating changes in family dysfunction scores to be unlikely due to maturity or differences in intelligence. If further research confirms the putative association of change in family drawings to the intervention, they might offer a novel and inexpensive way of documenting broader family change that may be relatively robust against demand characteristics, low verbal skills of younger children, and other limits to accurately measuring change using multi-informant, multi-format methodologies.

Inspection of the subscales of the FDP (as opposed to the overall family dysfunction score), showed that significant improvements over time were evident across a range of variables, including family pride, vulnerability, emotional distance, tension and anger, and global pathology. It might be argued that these scales represent core aspects of family functioning and are more straightforward and susceptible to improvement compared to other scales of vitality, role reversal, role exaggeration, and bizarreness, for which no change was found. It may also be that the constructs measured by these subscales are not generally particularly relevant to families of children with persistent conduct problems, but are more relevant for children with other problems, or for families in which more dysfunction is evident.

It should be noted that our use of the FDP entailed some modification from the original in that all family members could participate in the drawing. Our coding of contributions to the drawings indicated that the extent of adult involvement in the drawing process was not a confounder, however, it should be recalled that there was a slightly higher percentage of content estimated to be drawn by the children at follow-up. No significant correlations were found between the number of siblings and the child contribution to the drawing, confirming that the primary contribution was likely made by the target child. While we speculate that this could indicate that children became more cooperative following treatment and thus, readily participated in the task at follow-up, further research is needed to more carefully control for this factor. We found no associations between child involvement and other treatment variables, however, it is not entirely clear what role child and parent involvement played and it would be useful for studies to look at the relative merits of child alone vs. family-wide involvement in the FDP as a measure of treatment outcome and process.

Complementing previous research on the FDP (Wagner et al. 2015), children with high CU traits and their families displayed higher levels of representations of family dysfunction prior to treatment. This group also showed significantly more change on several aspects of family dysfunction (family pride, emotional distance, parental team, tension and anger), in comparison to the low CU group. Six months after the end of treatment, no difference between the two groups was found, displaying similar levels of representations of family dysfunction. Hypothesis b stating that children with high CU traits and their families would evidence higher levels of representations of family dysfunction was thus partially supported. This complements findings by Wagner et al. (2015) who examined this association with children’s internal representations, as opposed to collaborative representations, at one time point only and not in relation to an intervention. Further research using appropriate controls is needed to confirm this new finding of greater change in FDP dysfunction for families of children with high CU traits, to confirm it is a robust phenomenon reflecting real change in the family and not wholly stemming from the initially high scores.

Although overall the sample showed improvement on representations of family dysfunction over time, analyses revealed that this effect was largely driven by the high CU traits group, while the low CU group remained relatively stable. It has been put forward by Wagner et al. (2015) that the dimensions of relational functioning as measured by the FDP may be less related to overt behaviours such as conduct problems than to the emotional/affective processes that characterize callous-unemotional traits. Our results might be interpreted as suggesting that internal representations of family relations constitute a treatment mechanism that is more dominant in families with children who present with high CU traits. Strong, positive representations of family relationships may be particularly important to behavioural change in children with high CU traits and their families.

Reduced emotional distance (i.e., increased warmth) within the family pictures was more strongly related to reduced conduct problems in high CU compared to low CU children, further adding support to the notion that parental warmth is of unique importance to the development of children with CU traits (hypothesis c). In contrast, findings of previous studies reporting that enhanced parental discipline is more conducive to change in children not demonstrating CU traits could not be replicated (hypothesis d). No differential effects of callous-unemotional traits on family disorganization (e.g., task completion, rule compliance) were detected. Additionally, family disorganization did not significantly improve with treatment. As our measure was based on the family drawings which primarily tap into relational processes, it is likely that our measure did not effectively represent the construct of family organization/discipline.

Counter to expectation, no significant difference was found for tone of language of the one-word descriptors that families assigned to each family member. Tone of language did not become more positive in response to the intervention, hence refuting our final hypothesis (e). As reported above, the descriptors were found to coincide with some aspects of family dysfunction at follow-up, but no agreement was found before treatment. Indeed, tone of language of the descriptors observably started out rather positive, which did not match the drawings initially evidencing high dysfunction. On the other hand, positive tone of the descriptors at follow-up corresponded better to the improved family functioning depicted in drawings. A possible explanation for the present finding is that looking at families’ verbal communication is a more direct but also more superficial avenue to investigate family functioning, whereas drawings in their nonverbal nature may represent a more indirect and meaningful way of assessment. Words commonly chosen by families such as “nice”, “beautiful”, and “happy” appear positive but provide only a glimpse of family communication and are more likely to be suggested by parents than by children. This may not reflect actual family dynamics, which are usually complex and not straightforward, in the way that the Family Drawing Paradigm captures representations of family functioning.

### Limitations

Several limitations of this study warrant consideration. First and foremost, the absence of a randomized control design necessitated a focus on individual differences in treatment outcome rather than broader treatment efficacy. The lack of a control group restricts the interpretation that changes on the family drawings are due to the intervention. On a positive note, however, effect sizes for change in family dysfunction were large and were not associated with changes in intelligence or maturity in the children. Moreover, other studies that have led to important new leads in the field have featured similar within-group designs (e.g., Granic et al. 2007) and we feel it is important to present these data as a starting point for future research using family drawings. Second, the new modifications to the FDP’s rating system and its application to families have not been validated, and the variable and unknown numbers of participating family members in the drawing task may have introduced some unexplained variance. Third, the age range of included children was large, while family drawings are recommended as an assessment of attachment representations specifically in middle childhood. It is suggested for future studies pursuing this line of research to include a control group when comparing pre-post treatment effects on the FDP, using a sample of children in middle childhood. In addition, it might be informative to observe family interaction during this task to obtain alternative measures of family dynamics and organizational processes.

The results from this study are remarkable because they show that the effects of an evidence-based family intervention are not only noticeable by traditional methods such as questionnaires or behaviour observation, but are also reflected on a very different medium—family drawings. Children in parent-training interventions are usually too young to give their own accounts, and their perspective tends to be neglected. Seeking information from them through drawings can provide a way to hear children’s voices. In this regard, the Family Drawing Paradigm has proven a useful tool to capture subtle changes in children and their families’ representations of family functioning for research but undoubtedly also clinical purposes. It has been shown to tap into these changes that were not captured by self-report verbal descriptors, indicating added value of implicit measures such as drawings over and above self-report measures. Concerning callous-unemotional traits, the current findings strongly underline the importance of broader family processes and warmth in the parent-child relationship and their promotion in parent training interventions for these children. There is reason to be optimistic about the treatability of children with callous-unemotional traits, given an effective focus on these family processes.
